# Supplementary figures and images for: High diversity of fungal ecological groups from ice-free pristine and disturbed areas in the Fildes Peninsula, King George Island, Antarctica
Source: PLoS One. 2025 Jan 22;20(1):e0317571. doi: 10.1371/journal.pone.0317571 (PMC11753637; doi:10.1371/journal.pone.0317571)

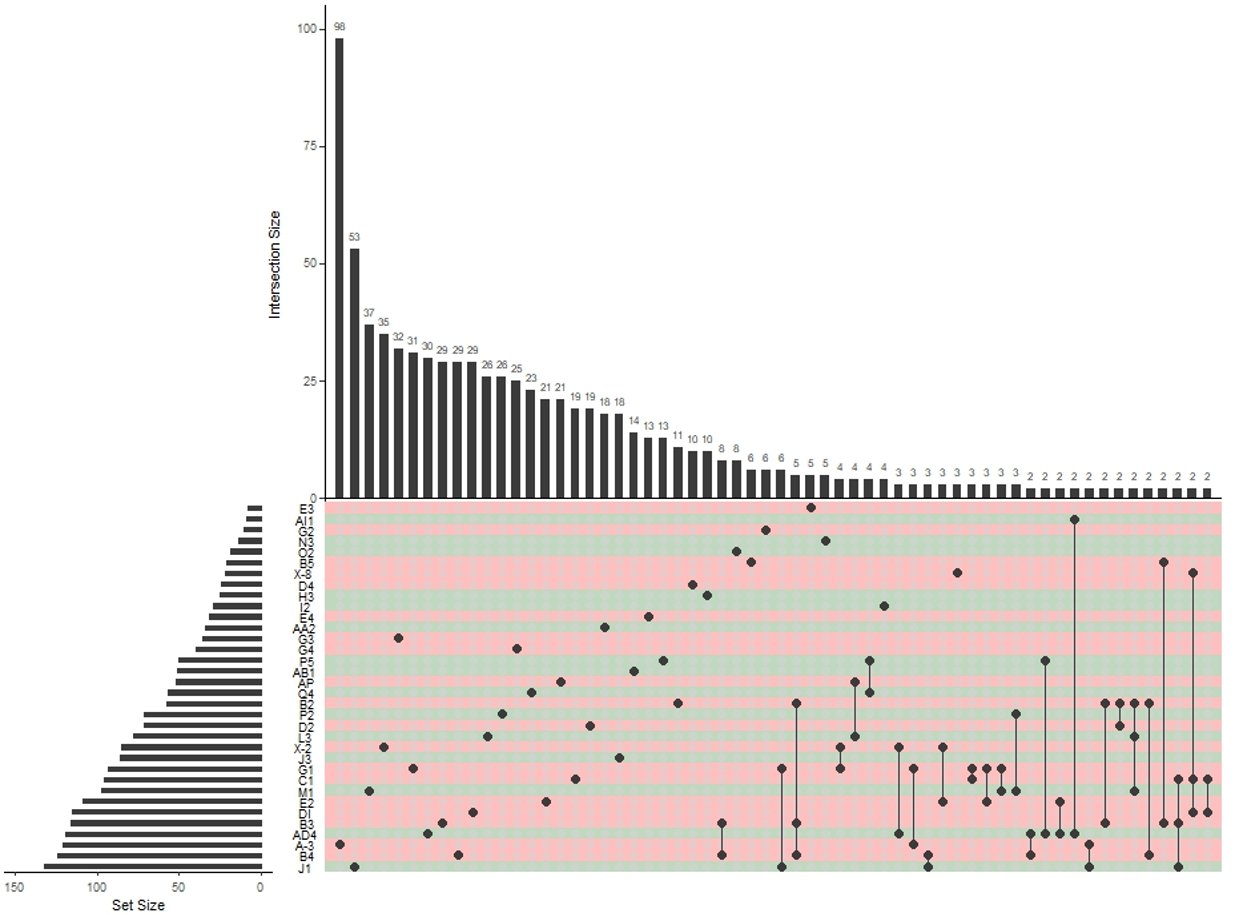

Supplement: S1 Fig — Relationships between reads and sediment samples collected at sites with and without anthropogenic impact are shown. (PNG) [file pone.0317571.s005.png]
